# Feasibility and Acceptability of VideoChat During Meals in Adults Aging in Place of Average Age 88

**DOI:** 10.1093/geroni/igab046.3604

**Published:** 2021-12-17

**Authors:** Alexa Lyman, Laura Barre

**Affiliations:** Cornell University, Ithaca, New York, United States

## Abstract

Older adults in the United States prefer to age-in-place. However, living and eating alone are risk factors for malnutrition. Using videochat during mealtimes, i.e., VideoDining, can provide commensality and social facilitation to improve nutritional intake. The objective of this study was to determine if older adults aging-in-place can independently VideoDine with family or friends. We recruited eleven older adults from Full Circle America Steuben, a virtual assisted living program for adults aging-in-place in rural New York. All participants had Amazon EchoShow devices for videochat. Participants were instructed on VideoDining and asked to independently schedule four VideoDine sessions with a family/friend in four weeks. Surveys were collected at baseline, after VideoDine sessions, and end-of-study. Participants were 91% female and 100% white. The average age was 88 years. All participants were widowed and living alone except for one married couple. Overall, 45% of participants VideoDined four times, 36% of participants VideoDined two to three times, and 27% not at all, for an average of 2.7 sessions in a month. Participants VideoDined during all meals, although dinner was most common (66% of meals), and breakfast least common (12% of meals). Average comfort was rated 7.6/10 (1=not comfortable, 10=comfortable), median enjoyment was 9.3/10 (1=not enjoyable, 10=enjoyable), and median ease of VideoDining was 4.1/5 (1=very difficult, 5=very easy). On average, participants rated their VideoDining meal experience a 7.6/10 (1=poor, 10=excellent). With access to videochat technology, older adults can connect with a dining partner and have a favorable experience sharing a meal over videochat.

